# Multiple Machine Learning Methods Reveal Key Biomarkers of Obstructive Sleep Apnea and Continuous Positive Airway Pressure Treatment

**DOI:** 10.3389/fgene.2022.927545

**Published:** 2022-07-13

**Authors:** Jie Zhu, Larry D. Sanford, Rong Ren, Ye Zhang, Xiangdong Tang

**Affiliations:** ^1^ Sleep Medicine Center, Department of Respiratory and Critical Care Medicine, Mental Health Center, West China Hospital, Sichuan University, Chengdu, China; ^2^ Sleep Research Laboratory, Center for Integrative Neuroscience and Inflammatory Diseases, Pathology and Anatomy, Eastern Virginia Medical School, Norfolk, VA, United States

**Keywords:** obstructive sleep apnea, machine learning, continuous positive airway pressure, bioinfomatic analysis, random forest (bagging) and machine learning, artificial neural network, SVM–support vector machine

## Abstract

Obstructive sleep apnea (OSA) is a worldwide health issue that affects more than 400 million people. Given the limitations inherent in the current conventional diagnosis of OSA based on symptoms report, novel diagnostic approaches are required to complement existing techniques. Recent advances in gene sequencing technology have made it possible to identify a greater number of genes linked to OSA. We identified key genes in OSA and CPAP treatment by screening differentially expressed genes (DEGs) using the Gene Expression Omnibus (GEO) database and employing machine learning algorithms. None of these genes had previously been implicated in OSA. Moreover, a new diagnostic model of OSA was developed, and its diagnostic accuracy was verified in independent datasets. By performing Single Sample Gene Set Enrichment Analysis (ssGSEA) and Counting Relative Subsets of RNA Transcripts (CIBERSORT), we identified possible immunologic mechanisms, which led us to conclude that patients with high OSA risk tend to have elevated inflammation levels that can be brought down by CPAP treatment.

## Introduction

The prevalence of obstructive sleep apnea (OSA) is estimated at one billion people worldwide, including over 400 million who have moderate-to-severe symptoms (Benjafield et al., 2019). The main characteristic of OSA is excessive sleepiness due to collapsed upper airways during sleep, resulting in oxygen desaturations, heart rate changes, neurological arousal, and therefore disturbed sleep (Young et al., 2004). In the absence of proper treatment, OSA contributes to a higher mortality rate from cardio- or cerebro-vascular events (Lévy et al., 2015). A variety of treatments have been developed to correct the narrowing of the upper airway in OSA patients. Continuous positive airway pressure (CPAP) treatment is the most extensively studied and proven therapy.

There has long been an indication that family history is a powerful risk factor for OSA. According to the Cleveland Family Study (Grilo et al., 2013), approximately one-third of the variance in the apnea-hypopnea index (AHI) can be explained by genetic factors shared across families. In addition, epidemiological evidence suggests a strong association between OSA susceptibility and genetic polymorphisms (Sun et al., 2015). Genes associated with cardiovascular consequences can be hypermethylated by hypoxia (Stenvinkel et al., 2007; Watson et al., 2010). Forkhead Box P3 (FOXP3), an immune-related gene, activates regulatory T cells and prevents atherosclerosis by modulating lipoprotein metabolism (Klingenberg et al., 2013). An increase in methylation of FOXP3 promoter has been linked to systemic inflammation among children with OSA. A strong correlation between DNA methylation levels and total C reactive protein (CRP) levels has been observed in OSA, suggesting a possible underlying mechanism (Kim et al., 2012). In fact, extensive inflammation is believed to be a major contributing factor to OSA. Multiple inflammatory biomarkers such as interleukin-6 (IL-6), tumor necrosis factor (TNF), CRP, and von Willebrand factor (VWF) antigen have been observed independently and consistently associated with OSA (De Luca Canto et al., 2015; Nowakowski et al., 2018; Hirsch et al., 2019). Nevertheless, the specific mechanisms that regulate this broadly activated inflammatory background remain unclear. Due to the recent emergence of next-generation sequencing, high-throughput techniques have enabled examining expression profiles for thousands of genes at a time. This has enabled identifying marker genes related to a wide range of diseases and has facilitated effective disease diagnosis and treatment.

As a result of excellent capabilities in handling large and complex datasets, artificial intelligence (AI) systems, which use multiple machine learning methods, have gained widespread popularity in evaluating genetic profiles. The recursive feature elimination (RFE) approach has demonstrated its effectiveness in selecting informative variables for disease classification. In order to aid in the identification of the least useful features to remove from consideration, a Support Vector Machine (SVM) classification model can be used to assign weights to features. Subsequently, each time the RFE procedure is executed, the least important feature, that having the smallest weight, can be eliminated thereby reducing the number of parameters and potentially increasing accuracy (Ding and Wilkins, 2006). The random forest (RF) algorithm is a method of reducing dimensions based on the creation of thousands of decision trees (Zheng et al., 2021). A random assignment of variables into testing and training groups is made first, followed by 10,000 iterations until the lowest error rate is achieved, and the optimal variable number and the optimal number of trees are determined. In addition, an RF model with variable importance values can be created (Bi et al., 2020). An artificial neural network (ANN), a method for supervised learning, consists of an interconnected group of artificial neurons arranged in layers (Alizadeh Savareh et al., 2020). An ANN is developed by changing weights of connection during the training phase to improve network performance.

Our primary aim in this study was to develop a novel prediction tool for OSA risk and CPAP treatment utilizing these three machine learning algorithms (SVM-RFE, RF, and ANN). We utilized transcriptome microarray data obtained from a public database, Gene Expression Omnibus (GEO). To establish machine learning models and discover key biomarkers associated with OSA and its therapeutic response to CPAP treatment, we integrated five independent microarray datasets related to moderate-severe OSA patients and their CPAP treatment. The results were also revalidated in another two separate datasets. Additionally, we assessed possible immunological mechanisms of OSA using multiple gene sets and enrichment analysis. As part of this research, we hoped to identify key genes that are implicated in OSA pathogenesis in addition to determining whether OSA is accompanied by dysfunctional innate immunity.

## Materials and Methods

### Data Collecting and Downloading

Patients with OSA, controls, and patients who had undergone CPAP treatment were included in this study. Genome profiles were derived from the GEO (Gene Expression Omnibus) database, which provides array- and sequence-based data. As the study utilized a public database, no approval from an institutional review board was required. A total of five microarray datasets were obtained. GSE133601 included 15 patients with moderate to severe OSA and who adhered to CPAP therapy over 3 months. Peripheral blood mononuclear cells were collected before and after CPAP treatment. GSE75097 involved 42 treatment-naïve subjects and patients with moderate to severe OSA that had received at least 1 year of adequate CPAP treatment. Peripheral blood mononuclear cells were collected. GSE71356 contained eight controls whose whole blood was collected. GSE61463 consisted of 16 OSA patients and five controls, and peripheral blood mononuclear cell samples were analyzed. GSE49800 was comprised of 18 subjects with severe OSA who had undergone CPAP therapy. Transcriptional profiles of peripheral blood leukocytes were assessed. GSE38792 and GSE135917 were used as independent validation sets. They provided information on subcutaneous and visceral adipose tissue transcription of OSA patients (including those who took CPAP treatment) and of controls.

### Data Processing and Batch Effect Control

R software (version 3.6.2) was used for statistical analyses. If multiple microarray probes were mapped to a single gene, its mean expression level was used in the analysis. The gene expression values were log2-transformed before normalization. All genes and samples were checked to ensure that no missing values were contained in the dataset. Quantile normalization was used to standardize data. The SVA package was used to conduct batch effect processing to remove differences caused by different platforms and technologies. Principal component analysis (PCA) was performed to verify whether or not the batch effect was eliminated. Genetic comparisons were performed between OSA patients and controls or between treatment-naive and CPAP therapy groups. Differential expression analysis was performed using the stringr and limma packages in R software. The fold change was derived from the average expression values. A logFoldChang (logFC) greater than 1.5 and *p*-value < 0.05 were set to identify the differentially expressed genes (DEGs). Continuous variables were compared between groups using equal variance. The significance threshold for the *p*-value was also set to <0.05.

### Gene Set Enrichment Analysis

GSEA analyses of RNA-seq profiles revealed DEGs-related signaling pathways in OSA patients and those who had undergone CPAP treatment. Screening of the enriched set was based on FDR (False Discovery Rate) < 0.25 and a *p* < 0.05 after 1,000 permutations. In GSEA, gene expression profiles from patient samples and controls were analyzed according to specific datasets (Subramanian et al., 2005). The GSEA website and the MsigDB database were used to obtain the c2. cp.kegg.v7.4. symbols.gmt dataset and c5. go.v7.4. symbols.gmt dataset for enrichment analyses, presenting Kyoto Encyclopedia of Genes and Genomes (KEGG) and Gene Ontology (GO) analyses for biologic pathways and function annotations. Statistically significantly enriched gene sets were defined as those with a minimum number of samples per group of 5.

### Cell-Type Identification by Estimating Relative Subsets of RNA Transcript Analyses for Infiltrating Immune Cells

CIBERSORT is a computational technique for quantifying cell fractions based upon bulk tissue gene expression profiles, which can distinguish 22 human hematopoietic cell phenotypes (Newman et al., 2015). It is widely applied in various diseases to accurately estimate underlying immunocellular landscapes. The CIBERSORT gene matrix contains 547 genes and distinguishes 7 T cell subsets, naïve and memory B cells, plasma cells, NK cells, and myeloid subsets (Wang et al., 2020). Heatmap analysis and correlation analysis of multiple immune cells in OSA patients and controls, or in those receiving CPAP treatment, were conducted using the pheatmap and corrplot packages, respectively.

### Single Sample Gene Set Enrichment Analysis Algorithm

ssGSEA identifies gene sets by their common biological functions, spatial localization, and physiological significance, and then it calculates separate enrichment scores for each pairing of a sample and gene set. Gene sets consisting of 782 genes are used for predicting the abundance of 28 types of immune cells and functions in individual tissue samples. The immune cells include activated dendritic cells (aDCs), B cells, CD8^+^ T cells, natural killer cells (NK cells), dendritic cells (DCs), neutrophils, macrophages, mast cells, plasmacytoid dendritic cells (pDCs), immature dendritic cells (iDCs), follicular helper T cells (Tfh), T helper cells, type-1 T helper cells (Th1), and type-2 T helper cells (Th2), tumor infiltrating lymphocytes (TILs), macrophages, regulatory T cells (Tregs). The immune-related functions consist of antigen presenting cell (APC) co-inhibition, APC co-stimulation, chemokine receptor (CCR), checkpoint, cytolytic activity, human leukocyte antigen (HLA), inflammation-promoting, MHC class I, para-inflammation, T cell co-inhibition, T cell co-stimulation, Type I IFN response, and Type II IFN response.

### Support Vector Machine Recursive Feature Elimination and Random Forest Algorithm

DEGs between OSA patients and controls or between treatment-naive and CPAP therapy groups were treated as variables in machine learning procedures. In the first step, SVM-RFE screening was performed for candidate genes. The SVM-RFE model employs a backward selection approach by which variables can be identified based on their weights on the model. A first ranking criterion is calculated using the SVM weights, then the features with the smallest ranking criteria are eliminated. The process is then repeated until the highest accuracy of classification is achieved.

Using the e1071 package and the svmRFE function in R software, we eliminated the recursive features of DEGs. All genes were sorted by their SVM weights in the linear SVM model, and those with low weights were eliminated. For the purpose of avoiding overfitting, a 15-fold cross-validation approach was used to increase the number of estimates. Fifteen subsamples from the original sample were randomly distributed. The model was tested using one subsample while the other subsamples were used as training data. After the 15-fold cross-validation was completed, the loop function provided an estimation of generalization accuracy and error rate. The best list of variables was determined based on the highest accuracy rate and lowest error rate. The Root Mean Square Error (RMSE) is defined as the standard deviation of the prediction errors, which measures the difference between the observed value and the actual value. The RMSEs were calculated from the 15-fold CV to verify the results of SVM-RFE.

A second machine learning algorithm, RF, was then applied to the candidate genes obtained from the SVM-RFE algorithm. It utilized a large number of decision trees and combined the bootstrap aggregation method to select features at random (Deo, 2015). Training data was collected for each tree through repeated subsampling (bootstrapping) (Hanko et al., 2021). Bootstrap subsamples excluding approximately 33% of the data provide an out of bag (OOB) sample (Hanko et al., 2021). The error rate was minimized by minimizing the number of decision trees included in the model, preventing overfitting. Internal validation of the RF was estimated using the OOB sample. The decreasing accuracy method was used to obtain the dimensional importance value based on the RF model. The genes with the highest importance values were selected for inclusion as variables in establishing an ANN.

### Construction of Gene Signature by Artificial Neural Network

R software neuralnet and NeuralNetTools packages were used to build an ANN model of the candidate genes screened by SVM-REF and RF (Beck, 2018). Typically, an ANN, another form of supervised learning algorithm, includes an interconnected set of artificial neurons in the form of intermittent layers.

There are typically three types of layers in neural networks: hidden, input, and output. Neurons are the basic components of computation, also known as nodes or units. A node receives inputs from other nodes or from outside and produces output after completing calculations. Each connection between two nodes represents a weighted value (W) for the signal passing through the connection. Each node applies a function f to the weighted sum of the inputs. The function f is nonlinear and is known as the activation function. In order to achieve the purpose of nonlinear representation of neurons and meet the data requirements of the actual environment, it introduces nonlinearity into the output of neurons. The function includes the inputs (x1, x2, … ), the weights (w1, w2, … ) associated with the inputs, and input weights (b): Output of neuron = f(w1.x1+w1.x2+…+b) . In each neuron, inputs are received at the previous layer, then output is sent to the next layer, and so forth until the output layer is reached. There is only one input layer and one output layer in a neural network, but there can be multiple hidden layers. Layers and neurons are not allocated according to a fixed rule. According to a broad consensus, one hidden layer can approximate any function that involves a continuous mapping from one finite space to another (Heaton, 2008). There are very few cases where a second or more hidden layer improves performance, and most of the time, one hidden layer suffices. The number of neurons in hidden layers are decided by input and output dimensionality. In cases where the input-output relationship is fairly simple, ideally, the optimal dimensionality for a hidden layer should be in the middle of the input layer and output layer (Heaton, 2008; Beck, 2018). In our ANN model, one hidden layer and five neurons were established for the classification model of OSA and CPAP treatment based on the principles outlined above.

A min-max normalization method was used to preprocess the data before training the neural network. Classification scores were calculated by multiplying the weight scores by the expression levels of the important genes. In a 5-fold cross-validation method, a training set and a verification set were randomly selected from the dataset. The training set served as the basis for determining the weights of candidate genes, while the verification set served as the basis for assessing the efficiency of classification. The R software pROC package was employed to assess classification accuracy.

## Results

### The Removal of Batch Effect Through Cross-Platform Normalization

**FIGURE 1:**
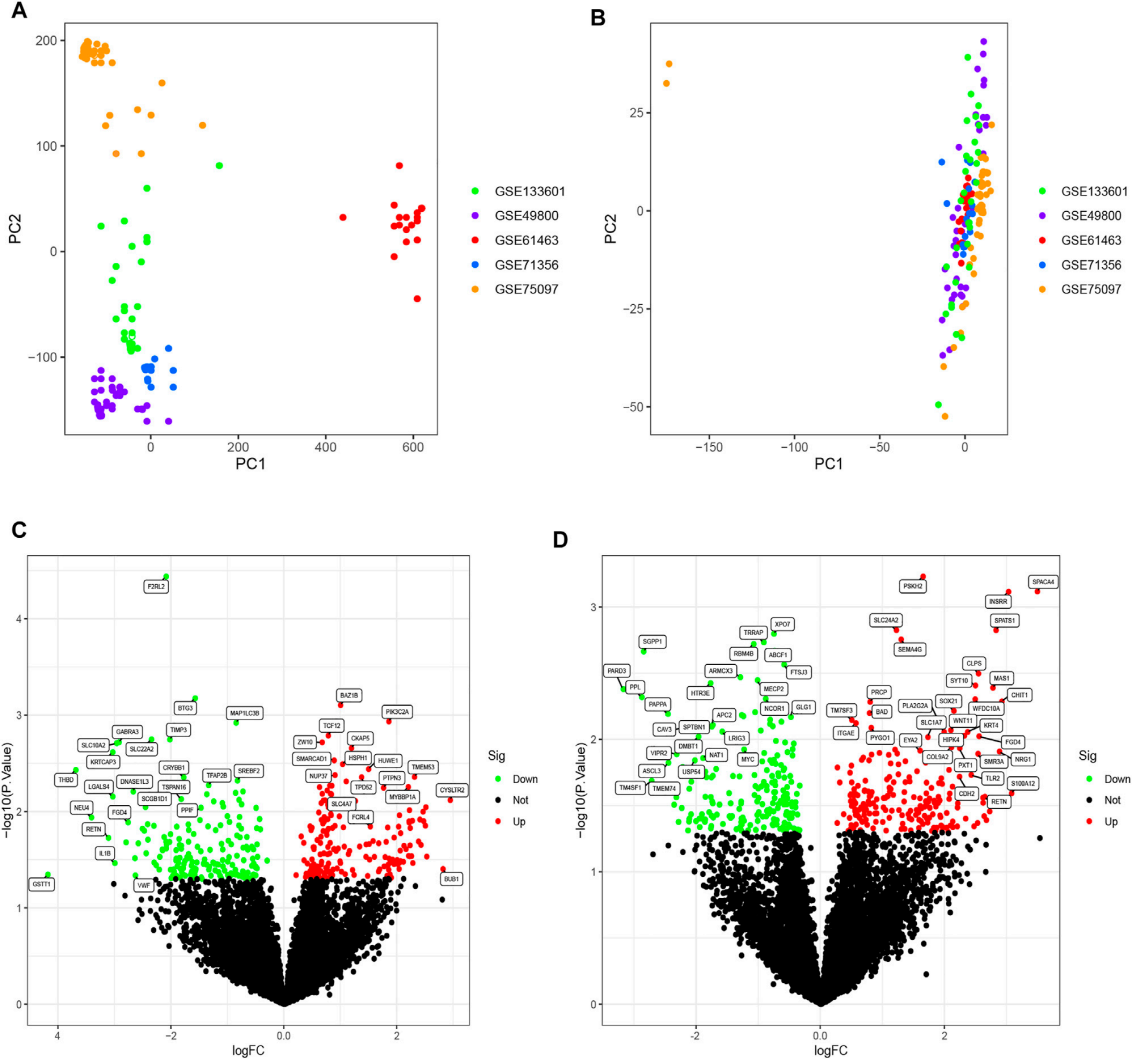
**(A)** PCA diagram before normalization. Samples from five datasets were distributed on both sides of panel A with a distinct boundary. **(B)** PCA diagram after normalization. After normalization, the data were tightly distributed. **(C)** Volcano plot of DEGs analysis in the OSA cohort. A logFC abscissa and **(A)** log10 *p*-value ordinate were used. Red plots in the upper right had a *p* value less than 0.05 and a fold change greater than 1.5, indicating up-regulated expression. Green plots on the upper left had a *p* value less than 0.05 and a fold change less than −1.5, indicating down-regulated expression. **(D)** Volcano plot of DEGs analysis in the CPAP cohort.

The R software ComBat function was used to eliminate batch effects due to non-ignorable technical differences across experiments, platforms, or studies. A total of 10,613 genes were detected in datasets from five different microarray platforms. Unnormalized and normalized PCA plots are shown in Figures 1A,B, respectively. Scatter plots illustrate the top two principal components (PCs) of expressed values. Unnormalized data plots indicate that the samples were loosely clustered and have distinct boundaries. As the samples clustered more tightly after normalization, they were more similar across datasets.

### Differentially Expressed Genes Analysis

Differential expression analysis was performed on two cohorts, between OSA patients and controls and between treatment-naive and CPAP therapy groups. The OSA cohort consisted of 77 OSA patients and 19 controls. The CPAP cohort comprised 47 individuals undergoing CPAP therapy and 61 treatment-naive OSA patients. Further, using the R software limma package, DEGs were identified between OSA and controls or between CPAP and treatment-naive samples. The results of the DEGs are presented in a volcano plot (Figures 1C,D). Based on fold changes >1.5 and significance thresholds of *p* < 0.05, 360 significant DEGs linked to OSA and 393 significant DEGs linked to CPAP were identified. A cross-comparison of two cohorts of DEGs identified 37 intersection genes: BAZ1B, MAP1LC3B, TCF12, HUWE1, TPD52, CRYBB1, PTPN3, BAD, CAND1, TXLNA, BHLHB9, GRPEL1, FGD4, REV3L, EXOSC10, SMAD4, TBX3, RETN, PPL, MGAT5, GLT1D1, SLC44A5, FAAH, FLT3, DOCK9, MGP, EPN3, TMEM121, ZNF214, CLEC10A, FKBP4, EYA2, MRO, TFF2, ABCF1, MOAP1, DNMT1.

### GSEA of DEGs

**FIGURE 2:**
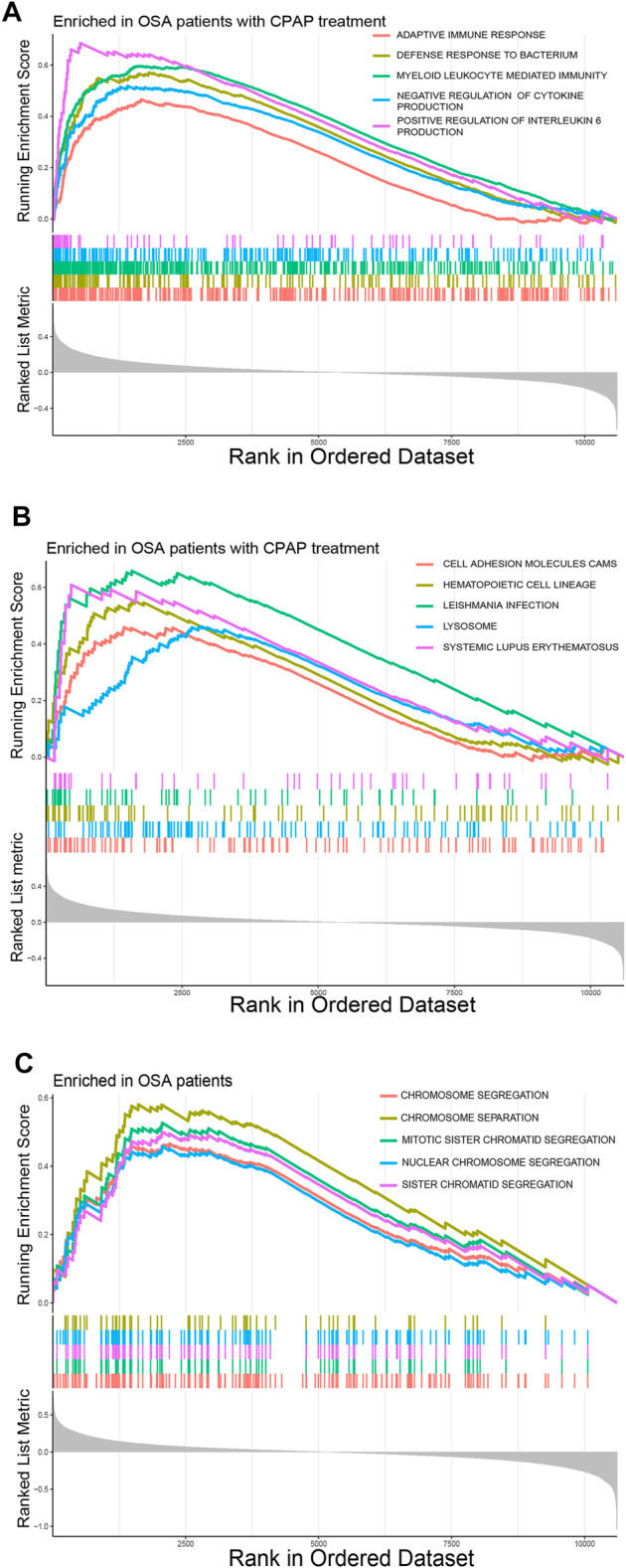
**(A)** Common pathways in OSA patients according to the GSEA c5. go.v7.4. symbols.gmt dataset. **(B)** Common pathways in OSA patients that had undergone CPAP treatment according to the GSEA c2. cp.kegg.v7.4. symbols.gmt dataset. **(C)** Common pathways in OSA patients according to the GSEA c5. go.v7.4. symbols.gmt dataset.

Some immune-related pathways were included in the GSEA results. DEGs of OSA patients who had undergone CPAP treatment were enriched in the adaptive immune response, defense response to bacterium, myeloid leukocyte mediated immunity, and negative regulation of cytokine production per the c5. go.v7.4. symbols.gmt dataset (Figure 2A). Several cellular structure-related pathways were also present in the results in OSA patients that had undergone CPAP treatment including cell adhesion molecules, hematopoietic cell lineages, and lysosomes per the c2. cp.kegg.v7.4. symbols.gmt dataset (Figure 2B). Some cell cycle-related activities were also observed in OSA patients, including chromosome segregation, mitotic sister chromatid segregation, nuclear chromosome segregation per the c5. go.v7.4. symbols.gmt dataset (Figure 2C).

### Selection of Candidate Genes and Construction of Predictive Signatures Using Multiple Machine Learning Algorithms Across Cohorts

**FIGURE 3:**
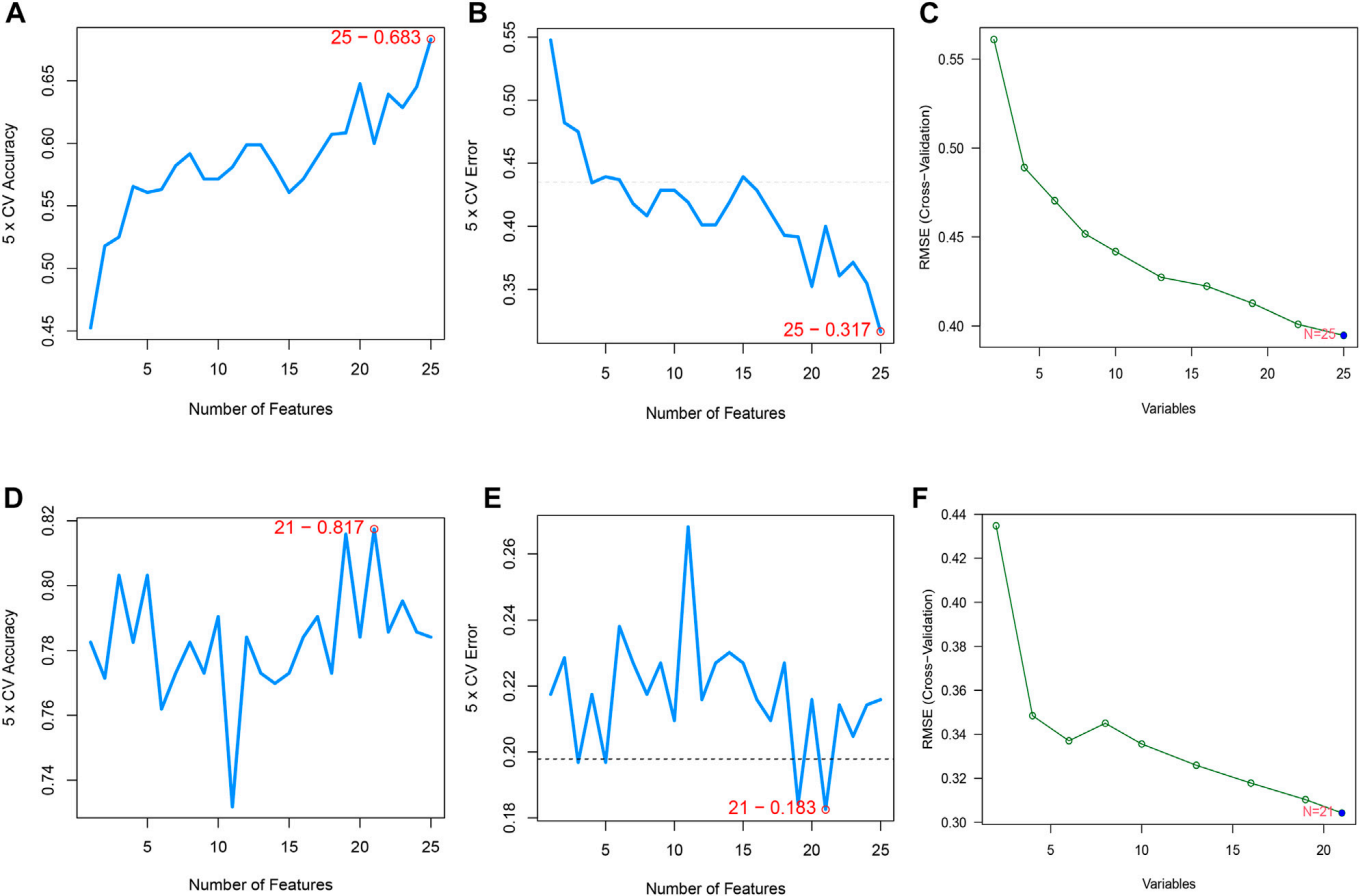
**(A,B)** Feature recursive optimization showing that the highest accuracy, and the lowest error, was achieved with 25 features (genes) in the OSA cohort. **(C)** The evaluation of RMSE in 15-fold cross-validation (CV) revalidated the results of SVM-RFE. **(D,E)** 21 features (genes) in the CPAP cohort were identified with the highest accuracy and lowest error obtained in the curves. The horizontal axis shows the number of feature selections based on CV, and the vertical axis shows the prediction accuracy **(F)** The RMSE was calculated from 15-fold CV and verified the results of SVM-RFE.

The SVM-RFE algorithm, which searches for genes with the smallest classification error, and RF, which detects genes with the highest importance, resulted in the selection of candidate genes. When the accuracy of the SVM-RFE algorithm was highest, and the estimation error was the lowest, 25 genes were identified in the CPAP cohort (Figures 3A,B) and 21 genes were identified in the OSA cohort (Figures 3D,E). We then input these genes into the RF classifier. By evaluating the RMSE, the best models were also determined to have a better balance of prediction errors (Figures 3C,F).

**FIGURE 4:**
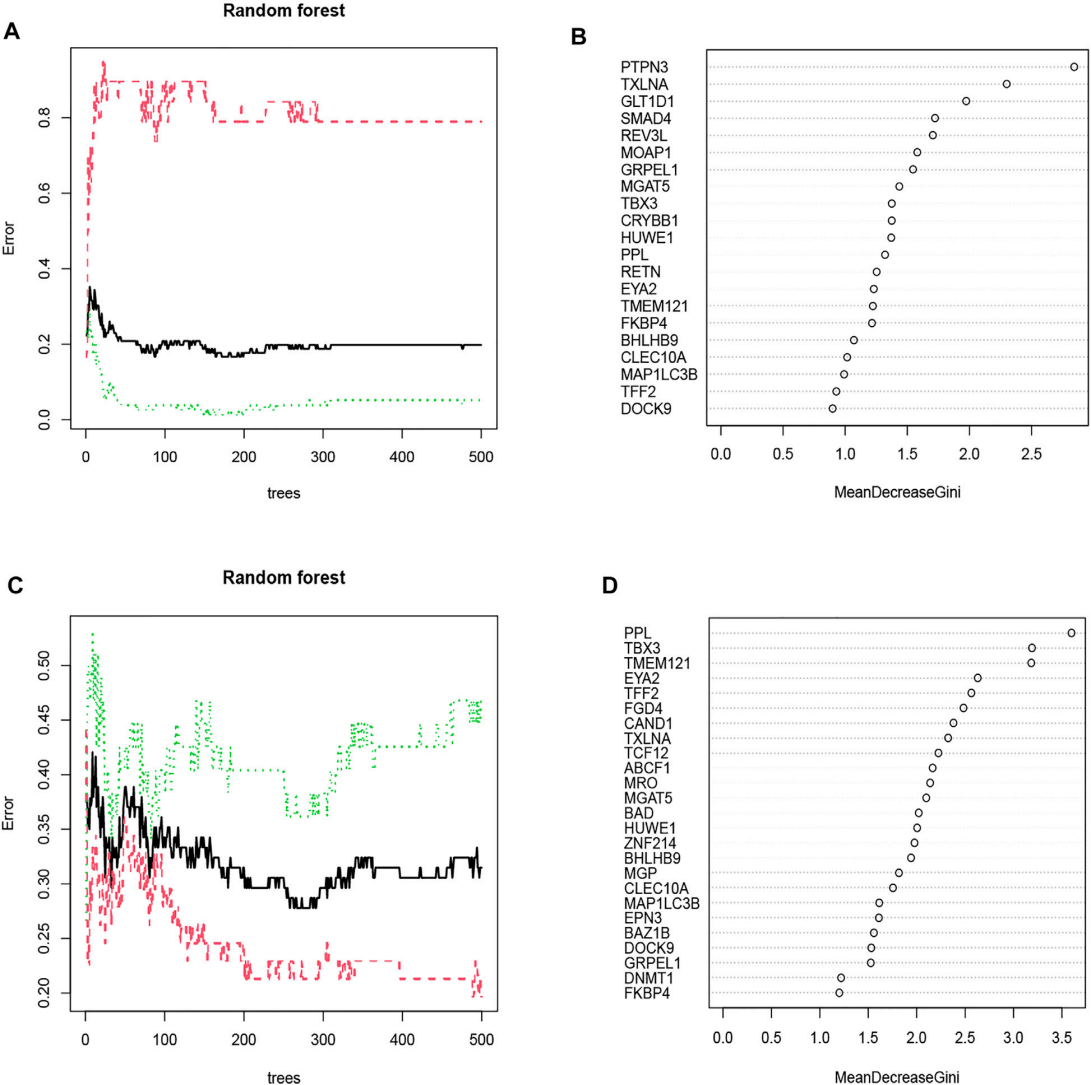
**(A)** The impact of decision tree number on error rate. The decision tree was plotted along the x-axis and an error rate along the y-axis. The OSA cohort’s error rate was relatively low when approximately 87 decision trees were plotted. **(B)** The Gini coefficient method in random forest modeling of the OSA cohort. Genetic variables are plotted on the y-axis, and importance indexes on the x-axis. **(C)** The CPAP cohort’s error rate was relatively low when approximately 258 decision trees were plotted. **(D)** The Gini coefficient method in random forest modeling of the CPAP cohort. Genetic variables are plotted on the y-axis, and importance indexes on the x-axis.

Each possible number of variables was analyzed with a recurrent RF classification to determine the average error rate. The error rate was relatively small when the number of decision trees was approximately 87 in the OSA cohort and 258 in the CPAP cohort (Figures 4A,C). Next, an RF model was built, and the Gini coefficient method was used to calculate the dimensional importance value. We chose the top ten genes with the greatest importance value as variables in each cohort’s subsequent construction of an ANN. In the OSA cohort, the top 10 genes were: PTPN3, TXLNA, GLT1D1, SMAD4, REV3L, MOAP1, GRPEL1, MGAT5, TBX3, and CRYBB1 (Figure 4B). In the CPAP cohort, the top 10 genes were: PPL, TBX3, TMEM121, EYA2, TFF2, FGD4, CAND1, TXLNA, TCF12, and ABCF1 (Figure 4D).

### Creating a Model of an Artificial Neural Network

The following formula was constructed to calculate the classification score for the ANN model: neural score = ∑(Gene Expression’ Neural Network Weight).

**FIGURE 5:**
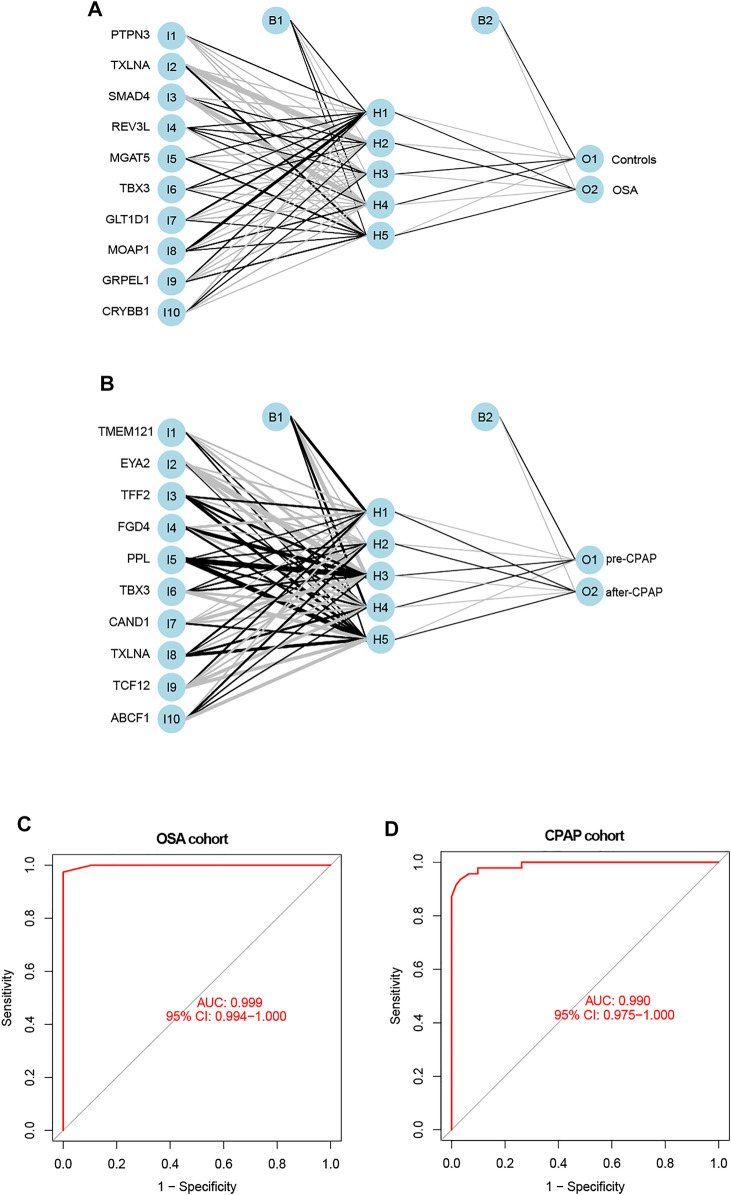
**(A,B)** The constructions of an ANN for the OSA cohort and the CPAP cohort were comprised of one input layer, one hidden layer, and one output layer. **(C)** ROC curves of the ANN-based OSA diagnostic model. **(D)** ROC curves of the ANN-based CPAP treatment model.

**FIGURE 6:**
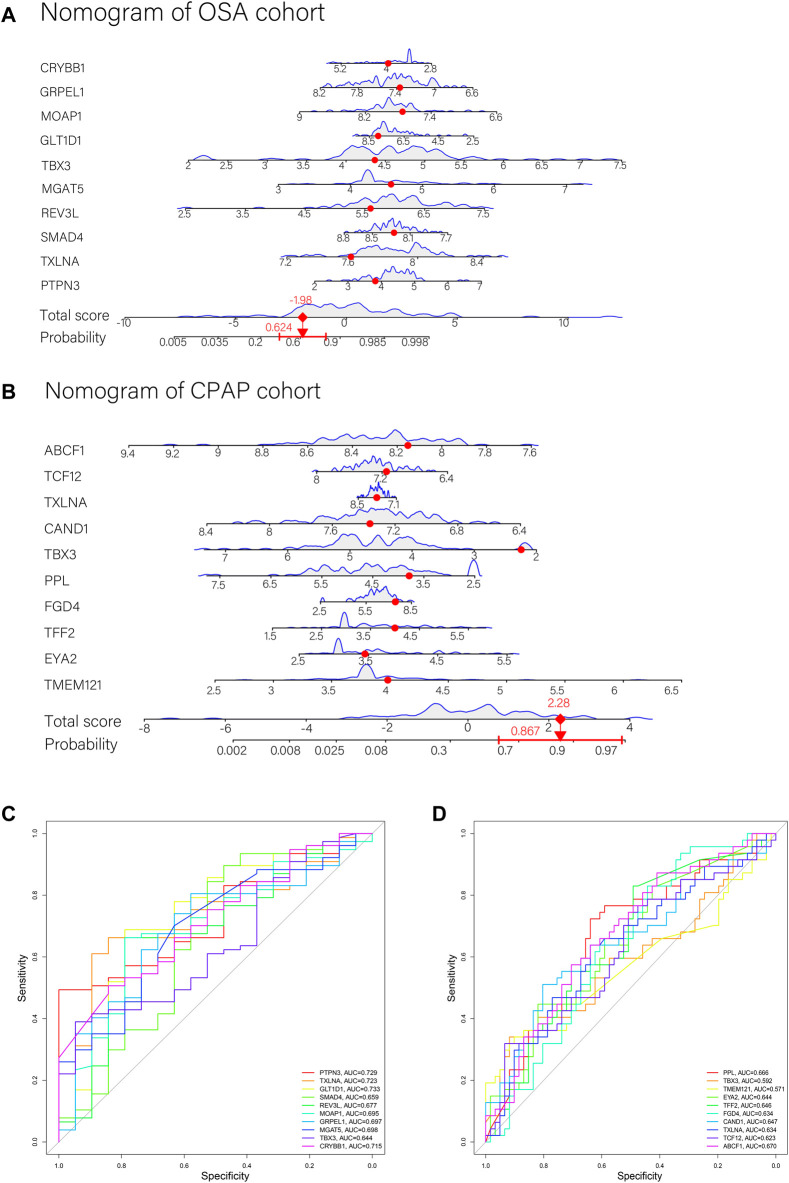
**(A,B)** Nomograms with key genes were constructed for OSA risk prediction and CPAP therapeutic response. A point line is shown on the horizontal axis for each variable. An axis for total score was plotted, and a line for probability was drawn downward to determine the risk or response to treatment. **(C)** ROC curves of ANN model genes in the OSA cohort. **(D)** ROC curves of ANN model genes in the CPAP cohort.

**FIGURE 7:**
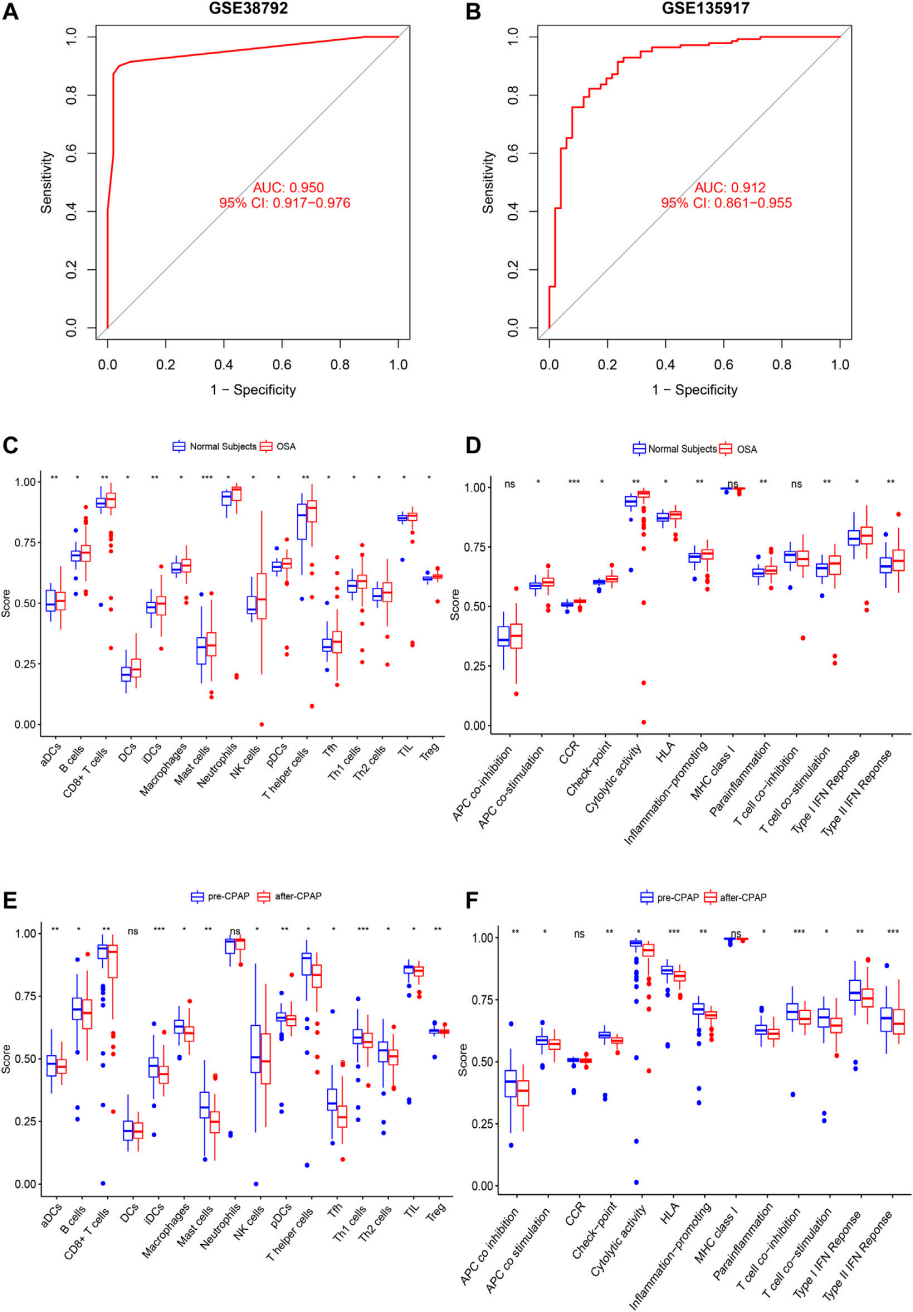
**(A,B)** Verification of the ROC curves by the ANN model for the GSE38792 and GSE135917 datasets. **(C,D)** The enrichment levels of 28 immune-related cells and functions in the ssGSEA results for the OSA cohort. Besides APC co-inhibition, MHC Class I, and T cell co-inhibition, other cell components, and functional pathways showed higher ssGSEA scores in OSA patients. **(E,F)** The enrichment levels of 28 immune-related cells and functions in the ssGSEA results for the CPAP cohort. Aside from DCs, Neutrophils, CCRs, and MHC class I, other immune cells and functions showed a downward trend in ssGSEA scores after CPAP treatment. *, *p* < 0.05; **, *p* < 0.01; ***, *p* < 0.001.

The weight predictions for the OSA cohort were 2.84 (PTPN3), -2.78 (TXLNA), -6.57 (SMAD4), 2.00 (REV3L), 1.41 (MGAT5), and 1.87 (TBX3), 17.77 (GLT1D1), 42.69 (MOAP1), 8.24 (GRPEL1), -7.96 (CRYBB1) (Figure 5A). Based on nomograms, MGAT5, REV3L, TXLNA, and PTPN3 were positively correlated with OSA risk, while the remaining six genes were negatively correlated (Figure 6A). The weight predictions for the CPAP cohort were -5.44 (TMEM121), -6.53 (EYA2), 8.75 (TFF2), -15.52 (FGD4), 2.54 (PPL), 1.72 (TBX3), -0.57 (CAND1), 8.66 (TXLNA), 7.99 (TCF12), -5.25 (ABCF1) (Figure 5B). Based on nomograms, the CPAP response was positively correlated with FGD4, TFF2, EYA2, and TMEM121, but was negatively correlated with other genes (Figure 6B). With the receiver operating characteristic (ROC) curve, the 5-time cross-validation illustrated the model classification performance. The areas under the curves (AUC) showed the hardiness of the model (average AUC >0.99) (Figures 5C,D). The AUC for each gene was also assessed within each cohort (Figures 6C,D). The AUC of the neural network score was much better than that of other genes. Furthermore, the same ANN model also had excellent performance across two independent validation cohorts from GSE38792 and GSE135917 (Figures 7A,B).

### Immune Cell Infiltration and the Neural Score

We used ssGSEA to examine immune infiltration in the transcriptomes of both OSA and CPAP cohorts, including twenty-eight immune-related terms to assess the abundance of immune cells.

**FIGURE 8:**
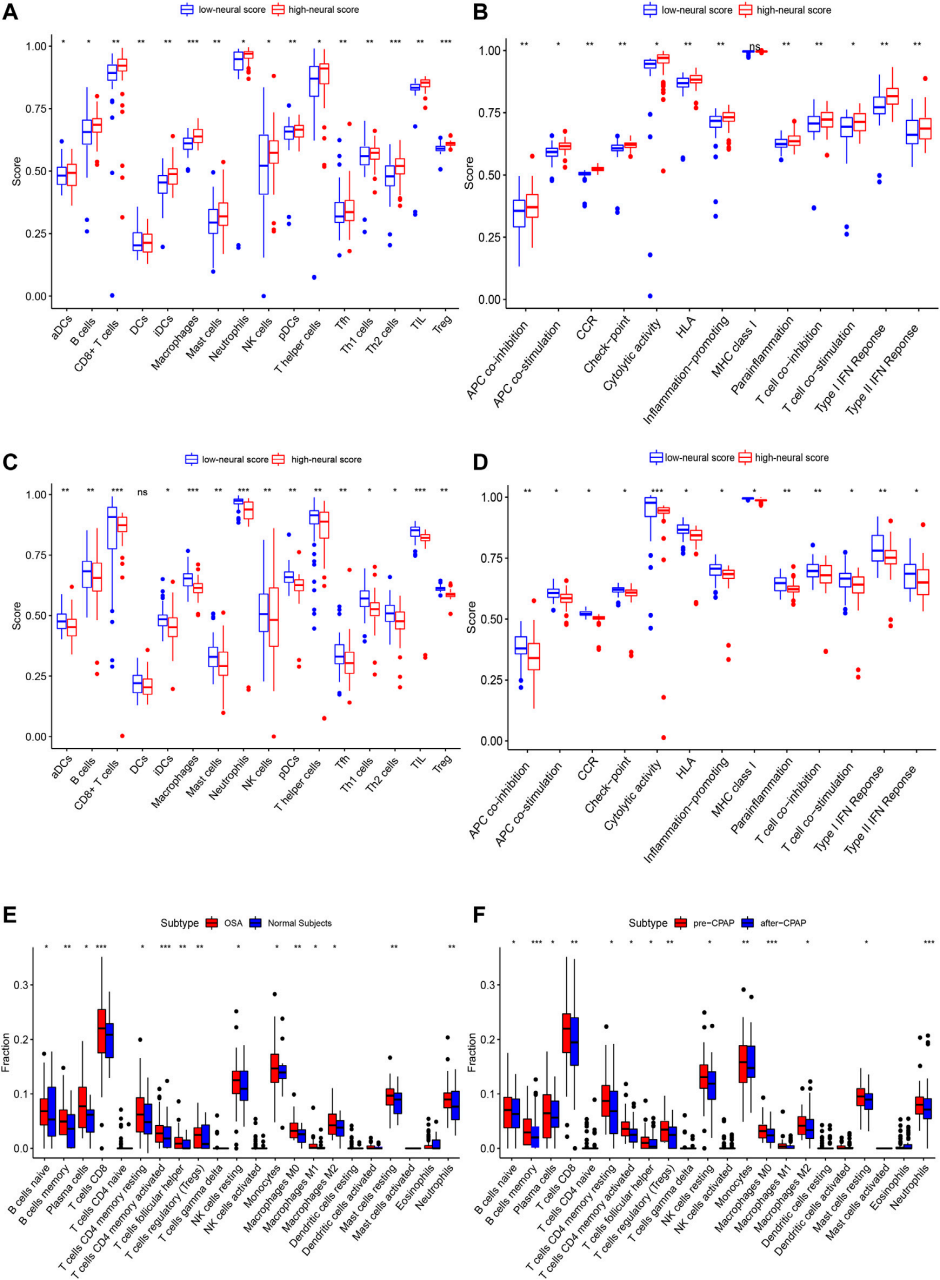
The enrichment levels of 28 immune-related cells and functions in the ssGSEA results for the ANN model. **(A,B)** Other than MHC class I, all other immune terms in the ssGSEA were increased in the high neural score group, suggesting that OSA is associated with increased inflammation. **(C,D)** In addition to DCs, other immune-related terms in ssGSEA decreased after CPAP treatment, suggesting that CPAP could reduce the level of inflammation in OSA patients. **(E,F)** CIBERSORT analysis of immune cell fractions of samples from OSA and CPAP cohorts. Patients with OSA had higher levels of inflammation in multiple immune cell components, which decreased with CPAP use. *, *p* < 0.05; **, *p* < 0.01; ***, *p* < 0.001.

In the OSA cohort, ssGSEA scores in multiple terms were higher in OSA patients than in controls, including diverse immune cells (DCs, B cells, T cells, macrophages, mast cells, neutrophils, NK cells, etc.) and a variety of immune pathways (APC co-stimulation, CCR, Checkpoint, Cytolytic activity, HLA, MHC class I, T cell co−stimulation, etc.) (Figures 7C,D). Almost all the elevated immune parameters responded to CPAP treatment. A decrease in the ssGSEA scores in immune cells and pathways was observed after CPAP treatment (Figures 7E,F). Our ANN model retained the differences in immunity between OSA and controls and before and after treatment with CPAP. The OSA patients were clustered into two groups based on the average neural score. Figure 8 shows the ssGSEA scores for the high-neural score and low-neural score groups. The high neural score group was associated with a higher ssGSEA score, indicating that a higher risk of OSA was associated with elevated immune infiltration (Figures 8A,B). Similarly, OSA patients with CPAP treatment were divided based on their average neural score. A higher neural score indicated a better CPAP treatment response, accompanied by lower immune infiltration (Figures 8C,D).

**FIGURE 9:**
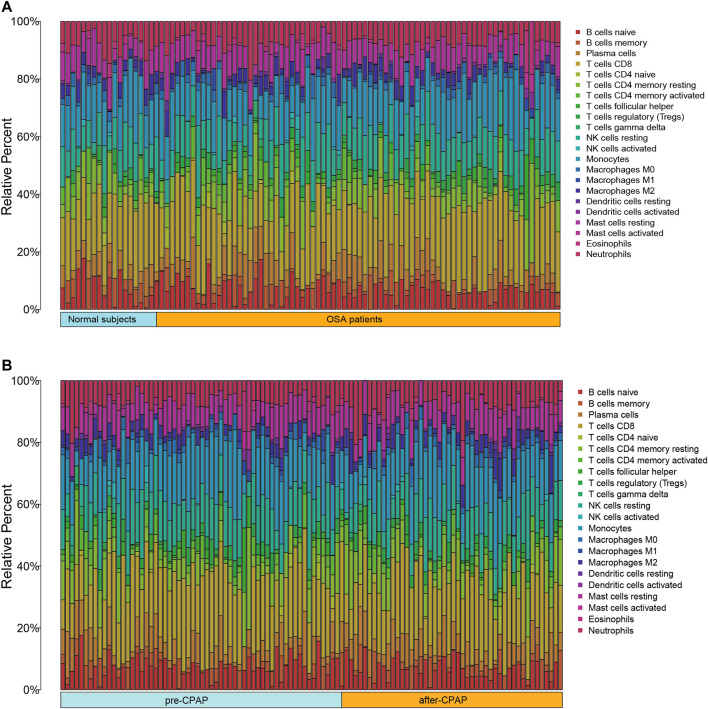
**(A)** Bar chart of 22 immune infiltrating cells comparing OSA patients and control samples **(B)** Bar chart of 22 immune infiltrating cells comparing OSA patients before CPAP and after CPAP treatment.

**FIGURE 10:**
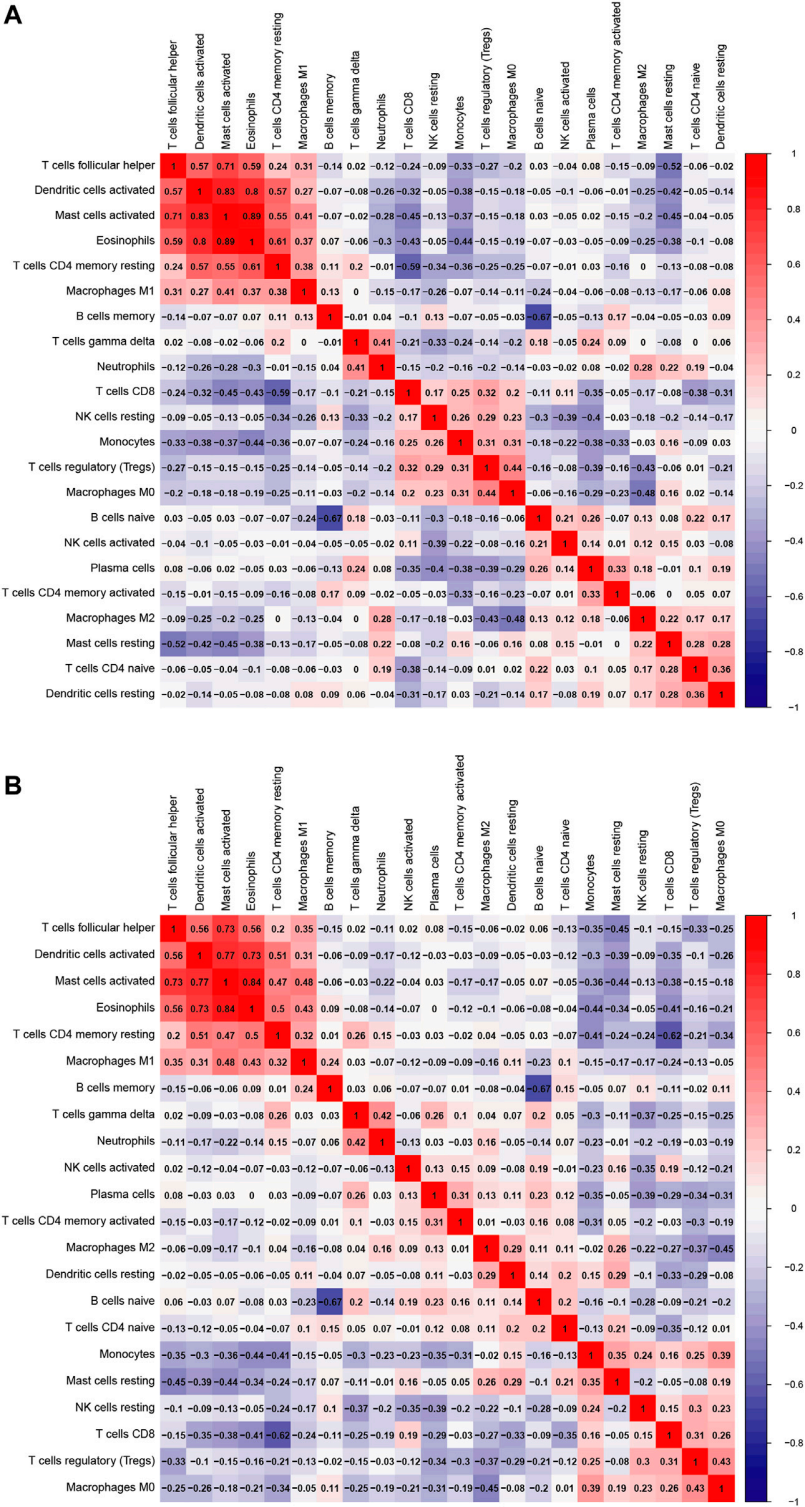
**(A)** Heatmap of 22 immune cells comparing OSA patients and control samples **(B)** Heatmap of 22 immune cells comparing OSA patients before and after the use of CPAP.

As a result of CIBERSORT, the proportion of 22 immune cell types in mixed tissue samples from the OSA and CPAP cohorts was estimated (Figures 9A,B). There were clear positive correlations among T follicular helper cells, activated mast cells, eosinophils, activated dendritic cells, and resting memory CD4 T cells (Figures 10A,B). Additionally, these immune cells exhibited negative correlations with monocytes, CD8 T cells, and resting mast cells. Diverse immune cells had a higher CIBERSORT fraction in OSA patients than controls (Figure 8E), such as B cells (naïve and memory B cells), plasma cells, T cells (CD8, resting CD4 memory cells, activated CD4 memory cells, etc.), NK cells, macrophages (M0, M1, M2), mast cells, and neutrophils. Additionally, nearly all types of immune cells showed decreased levels of elevation after CPAP use (Figure 8F).

## Discussion

OSA can be predicted using patient-reported symptoms such as sleepiness, snoring, and observed apnea (Holfinger et al., 2022). Questionnaires based on typical symptoms are widely used to assess OSA risk (Chung et al., 2008). However, such screenings tend to miss patients with mild symptoms and may include patients with conditions that cause similar symptoms. It is also believed that some physiological or pathological indicators, such as sex, age, BMI, smoking history, alcohol consumption, obesity, and hypertension, are highly associated with OSA risk (Gaspar et al., 2017; Peppard and Hagen, 2018; Drager et al., 2019). Furthermore, there is increasing recognition of the predictive role of inflammatory factors in OSA risk (Shamsuzzaman et al., 2002; Yokoe et al., 2003). However, there are no biological indicators to assess the risk of OSA that are as widely used in clinical practice as patient-reported symptoms. The development of high-throughput techniques has opened up numerous possibilities for gene-level analysis, providing an entirely new perspective on the assessment and treatment of OSA (Kim et al., 2012; Strausz et al., 2021). Significant advances made in targeting driver genes for cancer diseases suggests that new OSA prediction tools based on genetic data could contribute to identifying OSA risk and improving outcomes. Compared to traditional statistical tools such as logistic regression models, machine learning algorithms are more efficient at detecting multilevel, nonlinear relationships between variables and outcomes (Holfinger et al., 2022).

Machine learning also is rapidly being applied to various clinical models of diseases. According to Holfinger et al. (Holfinger et al., 2022), machine learning derived prediction tools based on age, sex, race, and BMI provide better diagnosis for OSA than logistic regression when used in community-based samples. Using sleep parameters and endoscopic findings to develop machine learning models for predicting the success rate of sleep surgery also showed higher accuracy than subjective prediction by sleep surgeons (Kim et al., 2021). Moreover, machine learning tools are effective at assessing long-term cardiovascular risk in OSA patients (Li et al., 2022). Machine learning has even been used to help build a CPAP compliance-monitoring system to improve the management of OSA patients (Turino et al., 2021). Additionally, machine learning is used to recognize data from polysomnograms (PSGs) and other monitoring equipment automatically. The data from PSGs is in the form of multichannel signals, making them ideal for machine learning techniques. The work of Linda Zhang has demonstrated how machine learning can assist in automating sleep staging and apnea/hypopnea detection, as well as building models to predict comorbid outcomes (Zhang et al., 2019; Zhang, 2021). As for other contactless devices, such as a microwave Doppler radar sleep monitoring system, machine learning is also capable of identifying OSA events (Snigdha et al., 2020). However, the performance of machine learning prediction models based on genetic datasets has not been examined. Utilizing three machine learning methods (ANN, RF, and SVM-REF), we performed a comprehensive analysis of transcriptome data for OSA risk and CPAP treatment.

Public databases were used to obtain microarray data on patients with OSA and those who had undergone CPAP treatment. Comparing OSA data with controls, including before and after CPAP treatment, DEG intersections were calculated. The final risk prediction model was comprised of ten genes and demonstrated excellent ROC performance as verified in multiple independent data sets. Several of these ten genes are involved in the immune system. PTPN3 is described as an immune checkpoint molecule. Increased expression of PTPN3 may act as a negative feedback mechanism that regulates the overactivation of lymphocytes and may be related to the PD-1/PD-L1 axis (Fujimura et al., 2019). PTPN3 has been implicated in various tumor studies as an immune system regulator and as an immunotherapy target (Gao et al., 2014; Peng et al., 2020; Koga et al., 2021). TXLNA, formerly known as interleukin-14 (IL-14), has been identified as a key factor in intracellular vesicle traffic, essential for cellular functions, such as neurotransmitter release, cell division, and cell motility (Nogami et al., 2003). It has been demonstrated that IL-14 promotes the proliferation of B cells and the expansion of memory B cells (Leca et al., 2008) and enhances the functions of memory B cells (Ford et al., 1995). SMAD4 has a critical role in activating TGF-β signaling pathways, a major immune-suppressive signal, affecting cytotoxic T cells and regulating the recruitment of regulatory T cells (Nakamura et al., 2001; Chen et al., 2003). In response to proinflammatory cytokines, SMAD4 proteins inhibit IFN-γ secretion by NK cells (Yu et al., 2006). MGAT5 encodes a glycosyltransferase called N-acetylglucosaminyltransferase V (GnT-V), required for T cell function (Daniels et al., 2002). GnT-V is tightly involved in regulating T cell activity and signaling. GnT-V deficient mice showed increased T cell receptor clustering, which led to a reduced threshold of T cell activation and increased TH1 differentiation (Demetriou et al., 2001; Morgan et al., 2004).

Several other genes are involved in important cell cycle and function processes. REV3L encodes the catalytic subunit of DNA polymerase zeta (Pol zeta), which belongs to the B family of DNA polymerases. A key function of this protein is to contribute to the tolerance of DNA damage by translesion synthesis (Prakash et al., 2005; Waters et al., 2009). By interacting with the BAX protein, MOAP1 serves as one of the key regulators of apoptosis, contributing to mitochondrial and death receptor-mediated apoptosis (Su et al., 2022). GRPEEL1 is a subtype of the GRPE protein homolog. It acts as a nucleotide exchange factor in mitochondria to influence nonnative protein folding (Ma et al., 2022).

The main characteristic of OSA is recurrent episodes of upper airway narrowing, which results in intermittent hypoxia (IH) and thus induces systemic inflammation. The activation of systemic inflammation and proinflammatory pathways are important mechanisms of OSA-derived chronic health conditions such as cardiovascular disease and cognitive impairment (Thompson et al., 2022). Widespread increases in inflammatory factors like TNF-α, interleukin 8(IL-8), Interleukin 6 (IL-6) (NF)-kB, and CRP levels have been observed in OSA patients and can be alleviated by CPAP treatment (Garvey et al., 2009; Kheirandish-Gozal and Gozal, 2019; Thompson et al., 2022). These proinflammatory factors are part of complex interactive networks generated by immune cells and vascular endothelial cells, adipose cells, and liver cells (Lee et al., 2007; Baessler et al., 2013). These factors were also highly variable among individuals, according to different studies (Kaditis et al., 2014; Gaines et al., 2016; Huang et al., 2016). It appears that genetic variances are responsible for similar heterogeneity (Riha et al., 2005; Popko et al., 2008; Kong et al., 2017). Given this background, finding important markers, particularly those closely related to OSA patients’ inflammation level and responding to CPAP treatment, will improve accuracy and provide new perspectives in identifying and treating these patients.

Accordingly, our ssGESA and CIBERSORT results provided similar conclusions. Multiple identified immune cells, such as B cells, T cells, plasma cells, NK cells, macrophages, and neutrophils, and diverse immune pathways were elevated in OSA patients and decreased after CPAP treatment. The predictive model based on machine learning algorithms maintained this characteristic, with individuals at high risk for OSA showing extensive activation of immune cells and pathways. Moreover, as mentioned previously, most genes in the models play an important role in the immune system. In addition, all ten genes were highly expressed in OSA patients, and their expression levels decreased after CPAP treatment. Furthermore, all patients in our study suffered from moderate to severe OSA. A majority of the studies reporting increased inflammation in OSA were based on moderate to severe cases (Murphy et al., 2017; Díaz-García et al., 2022; Popadic et al., 2022).

As the severity of OSA increased, so did the level of inflammation (Karamanli et al., 2017; Huang et al., 2021), which may have a more serious effect on gene transcription. These findings confirm the critical role that the immune background plays in developing and treating OSA and identifying important genes involved in OSA pathophysiology. There is little information regarding the relative roles played by these genes in OSA development. Further experiments *in vivo* and *in vitro* will be necessary to validate and examine the specific mechanisms involved.

While our results are promising, there also are several limitations to the current study. Machine learning based on genetic data poses challenges, especially regarding how to best apply results to clinical practice, given that genetic testing for OSA patients is not shared and costly. Second, AUC is a powerful tool for measuring model discrimination; however, its clinical utility is determined by the threshold met for sleep apnea treatment. A definite threshold has not been determined and remains subject to interpretation. Our models used a chosen cut-point to calculate predictive characteristics. Further population testing and model corrections and improvements are required before this type of analysis reaches its potential as a research and clinical tool for OSA.

## Data Availability

Publicly available datasets were analyzed in this study. The datasets can be found online (https://www.ncbi.nlm.nih.gov/). The names of the repository and accession number(s) can be found in the article.
